# Evidence of perturbed germinal center dynamics, but preserved antibody diversity, in end‐stage renal disease

**DOI:** 10.1002/iid3.108

**Published:** 2016-05-25

**Authors:** Kristian Assing, Christian Nielsen, Marianne Jakobsen, Alexandra Scholze, Mads Nybo, Grete Soerensen, Sussie Mortensen, Knud Vejen, Torben Barington, Claus Bistrup

**Affiliations:** ^1^ Department of Clinical ImmunologyOdense University HospitalOdenseDenmark; ^2^ Clinical Research UnitDepartment of NephrologyOdense University HospitalOdenseDenmark; ^3^ Institute of Clinical ResearchUniversity of Southern DenmarkOdenseDenmark; ^4^ Department of Clinical BiochemistryOdense University HospitalOdenseDenmark; ^5^ Department of NephrologyOdense University HospitalOdenseDenmark

**Keywords:** CXCL13, end‐stage renal disease, peripheral T follicular helper cells

## Abstract

**Introduction:**

End‐stage renal disease (ESRD) is associated with increased infectious susceptibility and with reduced vaccine responses consistent with compromised humoral immunity. Whether the compromised humoral immunity is due to reduced antibody diversity (reduced somatic hypermutation [SHM]) or altered germinal center (GC) dynamics is not known. The GC‐derived chemokine CXCL13 as well as peripheral T follicular helper cells (pTFH) reflect GC dynamics, but have, similar to SHM, never been characterized in relation to ESRD.

**Methods:**

Serum CXCL 13 was determined by ELISA. PTFH were flow‐cytometrically defined as CD4^+^ CD45RA^−^ CCR7^+^ CXCR5^+^ lymphocytes. Apoptotic lymphocyte subsets were in addition annexin V^+^. SHM was determined, by next‐generation sequencing and bioinformatics, as nucleotide mutations within the *IgG V_H_* (comprising the important antigen‐binding domains of IgG, *CDR1*, and *CDR2*).

**Results:**

Elevated CXCL13 levels characterized ESRD (*n* = 19; [median] 90 pg/ml, *P* < 0.01) (controls, *n* = 18; 62 pg/ml). ESRD pTFH frequencies (*n* = 19; 11.6% [of CD4^+^ memory T cells], *P* < 0.02*, *Bonferroni corrected) (controls, *n* = 22; 14.9%) and concentrations (*n* = 19; 0.03 × 10^9^/L, *P* < 0.02*) (controls, *n* = 22; 0.07 × 10^9^/L) were reduced. ESRD pTFH were more apoptotic (*n* = 9; 25.7%, *P* = 0.04*) (controls, *n* = 10; 15.9%). SHM did not discriminate between ESRD (*n* = 10; 7.4%, *P* = 0.21) and controls (*n* = 10; 8.4%).

**Conclusions:**

Elevated CXCL13 levels, reduced pTFH levels, and increased pTFH apoptosis suggest that perturbed GC dynamics, and not reduced antibody diversity, underlie the diminished vaccine responses and the compromised humoral immunity in ESRD. However, largely preserved SHM provides a rationale for pursuing vaccination in relation to ESRD.

## Introduction

End‐stage renal disease (ESRD) is characterized by attenuated responses to a range of vaccines 1, 2, 3, 4 and an increased propensity for infections with encapsulated bacteria 5, 6 consistent with compromised humoral immunity. Peripheral CXCR5^+^ T cells, comprising 20–25% of the CD4^+^ central memory T cell pool, are important for antibody‐recall responses 7. CXCR5 is the receptor for the germinal center (GC)‐derived chemokine CXCL13. Following activation, peripheral CXCR5^+^ T follicular helper cells (pTFH) support plasma cell differentiation and Ig production 8, and pTFH differentiation reflect GC function 9 In the GC, CXCL13 facilitates the interaction between CXCR5^+^ T and CXCR5^+^ B cells, resulting in the production of antigen‐specific antibodies 10. Progressively elevated CXCL13 levels relate to the antibody dysfunction accompanying progressive HIV infection 11 and correlate with disease severity and auto‐antibody production in SLE 12. The B‐cell compartment can be (severely) affected in ESRD 13. However, somatic hypermutation (SHM) may be more predictive of infectious susceptibility than reduced B‐cell numbers 14. It is, therefore, relevant to access SHM in ESRD patients, since decreased SHM will influence the range of microbes their humoral immune system can effectively engage. SHM entails the random insertion of nucleotides into the variable regions of heavy and light chain immunoglobulin genes 15 thereby ensuring additional antibody diversity. In the GC, SHM is followed by antigen mediated selection of high affinity antibodies 16 ensuring the best antibody fit to the eliciting antigen(s). Due to the aberrant vaccination kinetics and the increased susceptibility to infections with encapsulated bacteria, we hypothesized, that ESRD is accompanied by deviating CXCL13 and pTFH characteristics as well as reduced SHM.

## Materials and Methods

Preceding inclusion, participants received oral and written information and written consent was subsequently obtained from all participants.

### ESRD patients and controls

ESRD patients were maintained on hemodialysis (HD) for at least 6 months (three times/week) prior to inclusion (*n* = 19, protocol‐id: S‐20110085, clinical and para‐clinical characteristics, Table 1). ESRD patients with acute illness or in immunosuppressive therapy were excluded.

**Table 1:** Clinical and para‐clinical characteristics of ESRD patients and age/gender‐matched controls

	Controls (*n* = 22)	ESRD (*n* = 19)	*P*‐value
	Median (min–max)	Median (min–max)	Con. vs. ESRD
Age (years)	54.6 (33.3–67.2)	62.2 (30.3–82.1)	0.37
Women (%)	(45.5)	(52.6)	0.65
Creatinine (μmol/L)	70 (52–100)	818 (501–1,375)	<0.01
Blood urea nitrogen (mmol/L)	5.2 (3.5–8.7)	21.8 (7.1–29.9)	<0.01
CRP (<10.0 mg/L)	0.8 (0.3–8.2)	3.2 (0.5–50.8)	<0.01
Albumin (g/L)	47 (41–51)	39 (35−45)	<0.01
PTH (μmol/L)	4.6 (2.4–9.2)	38.7 (7.4–257.0)	<0.01
Leukocytes (10^9^/L)	6.3 (3.7–10.6)	6.6 (3.6–15.4)	0.92
Neutrophils (10^9^/L)	3.6 (1.7–7.3)	4.0 (1.8−13.4)	0.41
Monocytes (10^9^/L)	0.5 (0.3–0.9)	0.5 (0.3–0.8)	0.75
Lymphocytes (1^3^/L)	1.9 (0.8–3.1)	1.1 (0.9–1.9)	<0.01
CD3^+^ (10^9^/L)	1.3 (0.6–2.5)	0.9 (0.2–1.2)	<0.01
CD4^+^ (10^9^/L)	1.0 (0.4–1.9)	0.5 (0.2–1.0)	<0.01
CD8^+^ (10^9^/L)	0.4 (0.1–1.1)	0.3 (0.0–0.5)	0.26
CD19^+^ (10^9^/L)	0.2 (0.1–0.4)	0.1 (0.0–1.3)	<0.01
CD16^+^ CD56^+^ (10^9^/L)	0.2 (0.1–0.7)	0.2 (0.0–0.6)	<0.05
CD4^+^/CD8^+^	2.5 (1.1–9.8)	1.6 (0.6–6.0)	0.09
CD4^+^ CD45RA^−^ T cells (% CD4^+^)	42.3 (20.5–76.1)	64.2 (36.6–82.6)	<0.01

Among age‐ and gender‐matched controls (*n* = 22, protocol‐id: S‐20110085, clinical and para‐clinical characteristics, Table 1), pregnancy, infections, auto‐immunity, malignancy and chronic medication were causes for exclusion.

### Blood collection

Among controls, blood was collected by venipuncture by applying gentle aspiration. In the ESRD group, blood was drawn after canulation of the vascular access but prior to dialysis. In 11 ESRD patients, serum samples were also obtained immediately after dialysis. For the ESRD and the control group, leucocyte, neutrophil and monocyte counts were determined by the Sysmex XE‐1800i automated blood cell counter.

### T and B cells

Absolute numbers of CD3^+^, CD4^+^, and CD8^+^ T cells as well as CD19^+^ B cells were determined by flow‐ cytometry.

### Flow‐cytometric characterization of pTFH

Flow‐cytometrically, pTFH were identified as CD4^+^CD45RA^−^ CCR7^+^ CXCR5^+^ lymphocytes. Percentages of apoptotic CD4^+^ CD45RA^−^ CXCR5^−^ memory T cells and apoptotic CXCR5^+^ pTFH were determined by additional positivity for annexin V. Absolute pTFH numbers were calculated by multiplying their frequencies with the total CD4^+^ count.

**Figure 1:**
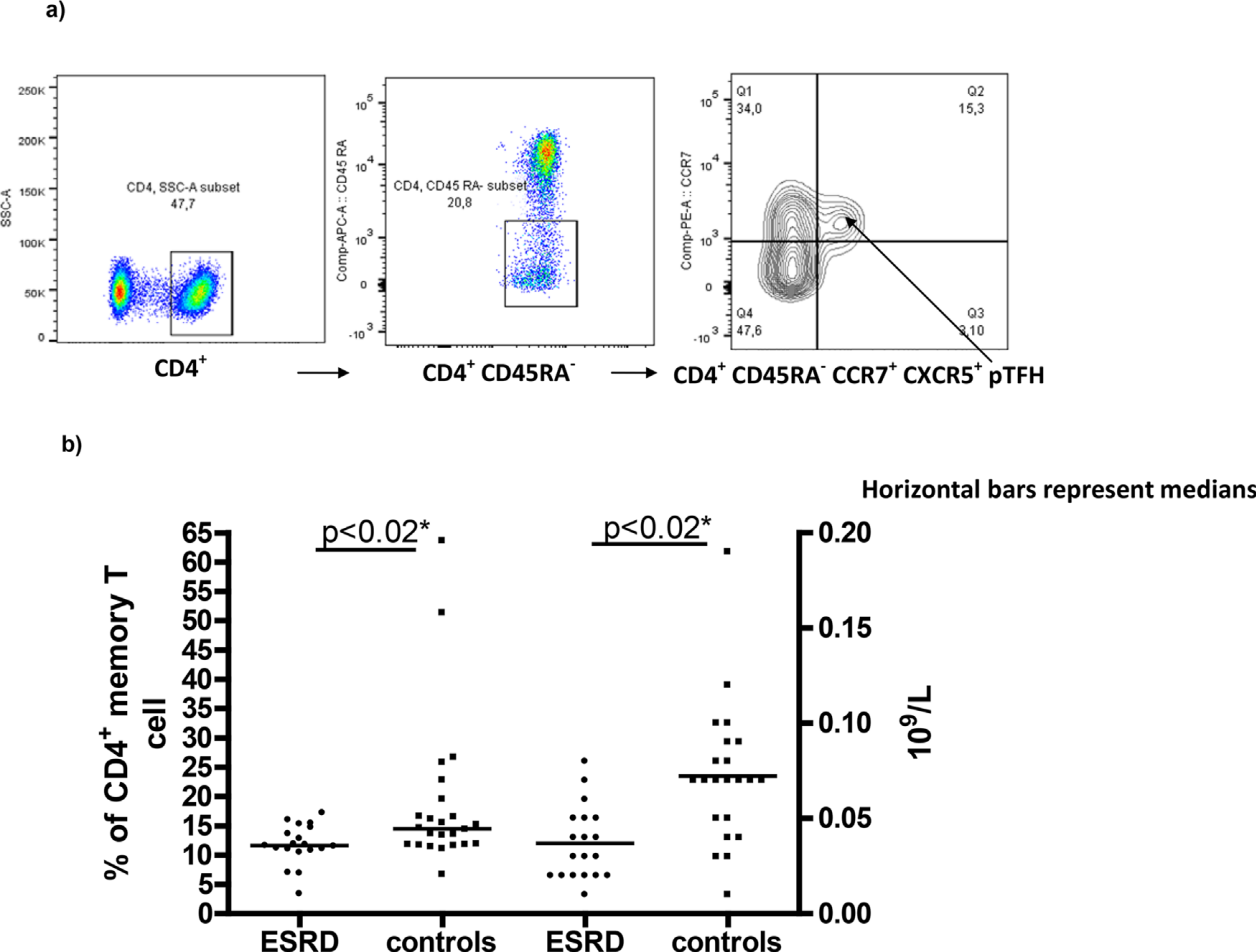
Peripheral T follicular helper cells (pTFH) in ESRD patients and controls. (a) Gating strategy for CD4^+^ CD45RA^−^ CCR7^+^ CXCR5^+^ pTFH. (b) PTFH frequencies (percentage of CD4^+^ memory T cells) and pTFH concentrations (10^9^/L) in ESRD patients and controls. Horizontal bars represent medians. *Bonferroni corrected.

Relative CXCR5 expression (delta [Δ] median fluorescence intensity [MFI]) was determined as: (MFI) (CD4^+^CD45RA^−^CCR7^+^ CXCR5^+^ subset) FITC channel—MFI (CD4^+^CD45RA^−^ CCR7^+^ CXCR5^−^ subset) FITC channel. The gating procedure was as follows: the initial gate was set on CD4 followed by a gate on CD45RA (as shown in Fig. 1a) followed by a gate on CCR7 followed by determination of CXCR5^+^ MFI respectively background MFI (FITC channel) of CXCR5^−^ central memory T cells.

### CXCL‐13 ELISA

Measurement of serum CXCL13 levels was performed with an enzyme‐linked immunosorbent assay (ELISA) (Quantikine Human CXCL13 ⁄BLC ⁄BCA‐1 Immunoassay; R&D Systems GmbH, Wiesbaden, Germany) using the manufacturer's instructions. Samples were run in duplicate.

### PCR and next‐generation sequencing of the IgG V_H_ gene

Mononuclear cells were thawed, and mRNA was isolated from 3 to 6 × 10^6^ cells using MagAttract Direct mRNA M48 kit (Qiagen, Hilden, Germany) on a KingFisher (Thermo LabSystems, Waltham, Massachusetts, USA). Synthesis of cDNA was performed in 1X PCR buffer II, 5 mM MgCl_2_, 1 U/μl RNnase inhibitor, 0.1 mM dNTP mix, 2.5 μM IgG specific primer (5′‐GCCTGAGTTCCACGACACC), 2.5 U/μl MulV reverse transcriptase (all from Life Technologies, Carlsbad, USA), and 51 μl of mRNA in a total volume of 100 μl. The reaction mixtures were incubated at 42°C for 30 min, and at 94°C for 5 min. The region of interest was amplified using the 0,3 μM 5′‐GTCCCTGAGACTCTCCTGT and 5′‐CTGAGGAGACGGTGACC in 1X Pfx amplication buffer, 0,3 mM dNTP mix in a total volume of 50 μl using 10 μl cDNA and 1 U platinum Pfx DNA polymerase (Life Technologies, Carlsbad, USA). The PCR was amplified with an initial denaturation at 94°C for 5 min, 40 cycles at 94°C for 15 sec, 5 °C for 30 sec, and 68°C for 60 sec.

The PCR products were prepared for next‐generation sequencing on the Ion Torrent PGM using the Ion Express Plus Fragment Library kit (Life Technologies). Ten to twelves samples were sequenced simultaneously with the 400 bp technologies using the Ion PGM Template OT2 400 kit, Ion PGM Sequencing 400 Kit and Ion 314 Chip v2 according to the manufacturers recommendations. Data were collected as FastQ files and converted to Fasta files. Data were filtered and reads containing reliably VDJ‐sequences were selected using a cluster analysis (PMID:25556246). The presence of V‐segment and number of SHM in these reads were determined using VDJsolver (PMID:17005006) 17.

### Analysis of nucleotide mutations within the IgG V_H_ gene (SHM)

The method has been published in Barington et al. 18: reads were qualified by the presence of primer and MID sequences and identical sequences (at least 1 forward and 1 reverse) were clustered. Clusters from the same PCR could be identified based on the primers and MIDs and were analyzed separately in the following: Clusters were evaluated by a numerical programming algorithm designed by the authors (details available from the corresponding author). In brief, clusters were ordered after declining number of members. The sequence of the largest cluster was accepted if the cluster had an acceptable balance of forward and reverse sequences. If not accepted, the algorithm continued to the next cluster. Once a sequence was accepted, it was compared with all smaller clusters from the same PCR and these were discarded if their sequence did not deviate by at least a certain number of substitutions or insertions/deletions (indels) from the index sequence. The number depended on the cluster sizes and was selected to account for Taq‐errors and other common sequencing artefacts that allowed clonal sequences to diverge slightly. When all smaller clusters had been dealt with, the process reiterated from the largest of the remaining clusters and this process continued until all clusters were either accepted or discarded. Rearrangements passing the cluster evaluation were analyzed using VDJsolver (http://www.cbs.dtu.dk/services/VDJsolver/) as previously described 17 with the following modifications: (i) improved identification of V_H_‐genes by a Smith‐Waterman algorithm comparing with a database of V_H_ germline genes compiled from the international ImMunoGeneTics information system (IMGT) (http://www.imgt.org) with omission of sequences unlikely to represent true germline genes (level 5) as reported by Wang et al. 19 (ii) improved sensitivity for D‐genes by lowering the penalty for inclusion of one D gene (iii) acceptance of two D‐genes in a rearrangement after accepting a penalty for two D genes.

### Statistical analysis

**Figure 2:**
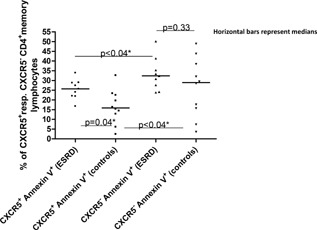
Apoptosis in CXCR5^+^ pTFH and in CD4^+^ CXCR5^−^ memory T cells among ESRD patients and controls. Annexin V positivity in (CD4^+^ CD45RA^−^) CXCR5^+^ (pTFH) and (CD4^+^ CD45RA^−^) CXCR5^−^ memory T cells derived from ESRD patients and controls. Horizontal bars represent medians. *Bonferroni corrected.

SPSS (version 11, Chicago, IL, USA) was used. Comparisons between multiple independent groups were performed by the Kruskal–Wallis test, followed by the Mann–Whitney *U*‐test (two independent groups with continuous values). In Figures 1 and 2(with multiple comparisons), significant results were Bonferroni corrected. Comparisons between two independent groups (binary values) were performed with the Chi‐square test. Paired analyses were performed with the Wilcoxon signed rank test. Correlations were performed with the Spearman's rank test. Values were presented as medians and minimum and maximum values. A two‐sided *P*‐value < 0.05 was regarded as significant.

As the number of unique *IgG V_H_* transcripts (each representing a unique hypermutated B cell) showed considerable interpersonal variation, we calculated the SHM value ([number of mutated nucleotides/number of investigated nucleotide positions within each unique *IgG V_H_* transcript] × 100) for each unique *IgG V_H_* transcript, followed by the calculation of a median SHM value (ranging over all unique *IgG V_H_* transcripts per participant) for each control and ESRD patient and finally followed by group comparison. A requirement for the determination of an individual median SHM value was: a) ≥ unique 10 *IgG V_H_* transcripts per person + b) that each unique *IgG V_H_* transcript contained >3 nucleotide mutations (in order to account as hypermutated). The frequency of non‐mutated ([number of unique *IgG V_H_* transcripts with ≤ 3 nucleotide mutations per transcript/total number of unique *IgG V_H_* transcripts per participant] × 100), GC‐derived, B cells was calculated for each ESRD patient and control and followed by group comparison.

## Results

### ESRD patients

ESRD (clinical data, Table 1) was secondary to hypertensive nephropathy in five cases; type I diabetes in four cases; polycystic renal disease in three cases; hydronephrosis in three cases; and single instances of TTP, juvenile nephronetosis, medullary sponge kidney, and nephrolithiasis. Compared with controls, the ESRD group was characterized by elevated creatinine, blood urea nitrogen, parathyroid hormone (PTH), and C‐reactive protein (CRP) levels but reduced albumin levels. ESRD patients resembled controls with regard to leukocyte concentrations apart from reduced B‐ and T‐lymphocyte subset concentrations and a tendency to reduced CD4^+^/CD8^+^ratios, Table 1. The percentage of CD4^+^ CD45RA^−^ (memory) T cells was increased in the ESRD group (*n* = 19; 64.2% (of CD4^+^ T cells); 36.6–82.6%) compared to the control group (*n* = 22; 42.3%; 20.5–76.1%), *P* < 0.01, Table 1. A mean K_t_/V value of 1.4 indicated the clinical sufficiency of the dialysis regimen.

### PTFH characteristics

Compared to controls (*n* = 22; 0.07 × 10^9^/L; 0.01–0.19 × 10^9^/L), ESRD patients (*n* = 19; 0.03 × 10^9^/L; 0.01–0.08 × 10^9^/L, *P* < 0.02, Bonferroni corrected) were characterized by reduced absolute numbers of pTFH (Fig. 1). PTFH frequencies (% of CD4^+^ memory T cells) also differed between controls (*n* = 22; 14.9%; 6.7–63.6%) and ESRD patients (*n* = 19; 11.6%; 3.4–17.2%, *P* < 0.02, Bonferroni corrected) (Fig. 1). Compared to those of controls (*n* = 21; 79 Δ MFI; 45–165 Δ MFI), pTFH, from ESRD patients, tended toward diminished CXCR5 positivity (*n* = 19; 64 Δ MFI; 13–112 Δ MFI, *P* = 0.12, data not shown).

### Annexin V and the CXCR5^+/−^dichotomy

A signature of the ESRD group (*n* = 9; median: 25.7%; 16.8–34.0%, *P* = 0.04, Bonferroni corrected) was increased frequencies of pTFH positive for the apoptosis marker annexin V 20 (controls: *n* = 10; 15.9%; 2.4–32.6%) (Fig. 2). Frequencies of CD4^+^ CD45RA^−^ CXCR5^−^ memory T cells, positive for annexin V, did not discriminate between ESRD patients (*n* = 9; 32.4%; 23.9–50.2%, *P* = 0.33) and controls (*n* = 10; 28.9%; 3.5–48.9%) (Fig. 2). For both ESRD patients and controls, CXCR5^+^ pTFH were less apoptotic than CD4^+^ CXCR5^−^ memory T cells (*P* < 0.04, Bonferroni corrected), (Fig. 2). Judged by forward‐side scatter characteristics 21, annexin V^+^ cells, from both ESRD patients and controls, were not dead.

### CXCL‐13 in ESRD patients and controls

**Figure 3:**
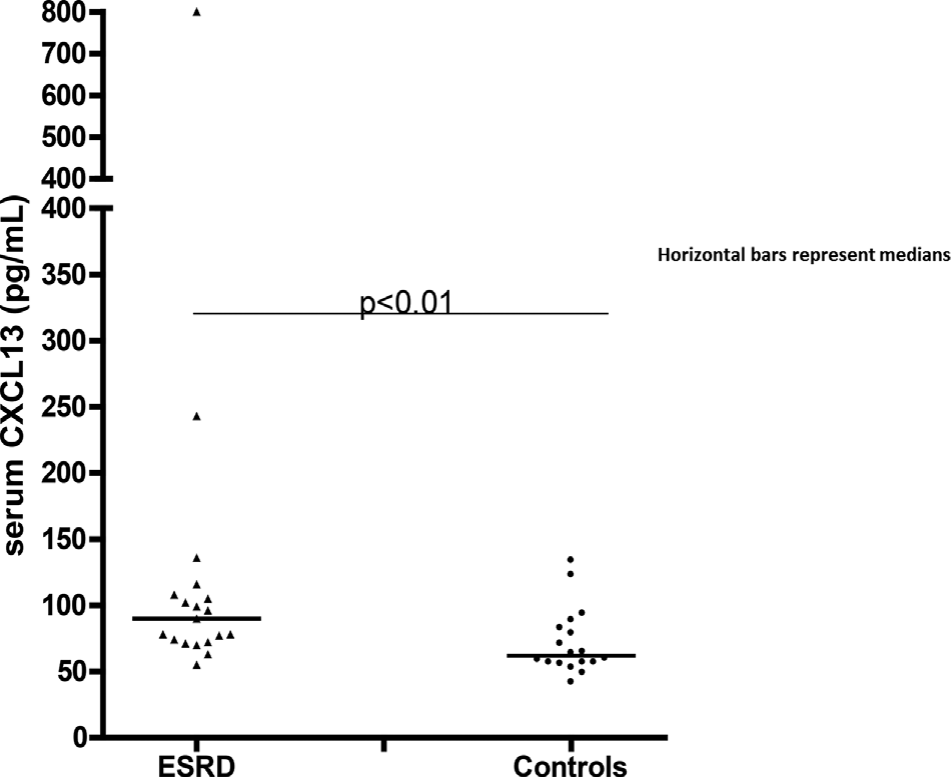
Serum CXCL13 levels in ESRD and healthy controls. Serum CXCL13 concentrations in the ESRD and control group. Horizontal bars represent medians.

CXCL13 levels were elevated in ESRD (*n* = 19; 90 pg/ml; 55–800 pg/ml, *P* < 0.01) versus controls (*n* = 18; 62 pg/ml; 42–134 pg/ml) (Fig. 3). CXCL13 levels in ESRD, censored for clinical autoimmunity (*n* = 14; 87 pg/ml; 55–243 pg/ml, *P *= 0.01), also differed from controls (data not shown). CXCL13 levels among ESRD secondary to clinical autoimmunity (four type I diabetes and one TTP) (*n* = 5; 90 pg/ml; 70–800 pg/ml) did not differ from ESRD patients without clinical autoimmunity, *P* = 0.85 (data not shown). In the ESRD group, CXCL13 levels did not discriminate between pre‐ (*n* = 11; 63 pg/ml; 44–106 pg/ml, *P* = 0.86, data not shown) and (immediate) post‐dialysis (*n* = 11; 70 pg/ml; 39–254 pg/ml), but pre‐ and (immediate) post‐dialysis levels correlated (Spearman, *n* = 11, ρ = 0.61, *P* <0.05).

### SHM

**Figure 4:**
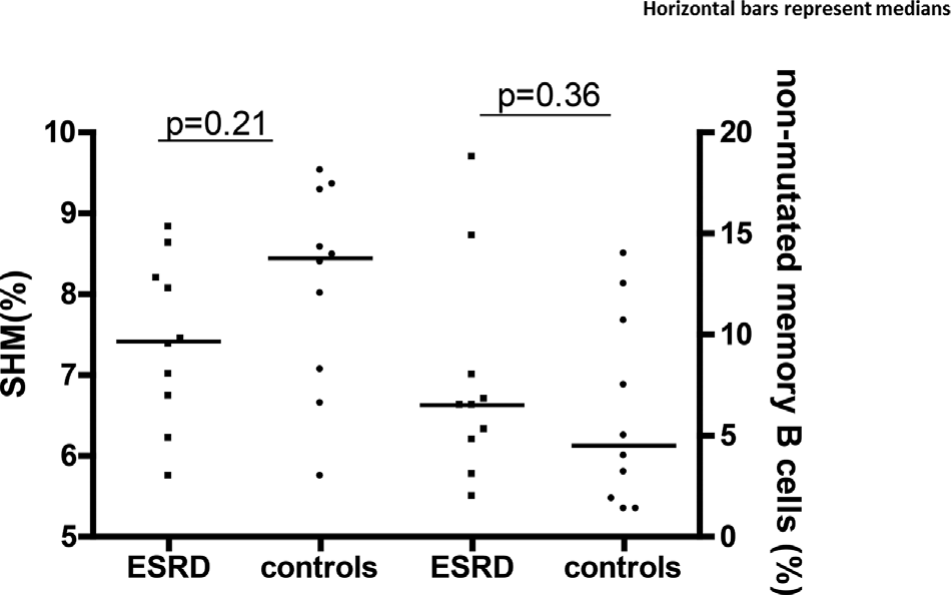
Minor differences in somatic hypermutation (SHM) between ESRD and controls. Somatic hypermutation (SHM) within the *IgG V_H_* gene (comprising the antigen‐binding domains CDR1 and CDR2) and frequencies of non‐mutated (expressing unique *IgG V_H_* transcripts with ≤ 3 nucleotide mutations per *IgG V_H_* transcript), GC‐derived, B cells in ESRD patients and controls. Horizontal bars represent medians.

The degree of nucleotide mutation within the *IgG V_H_* region (comprising the important antigen‐binding domains: complementary determining region ([*CDR*] *1* and *2*) did not discriminate between ESRD patients (*n* = 10; 7.4%; 5.8–8.8%, *P* = 0.21) and controls (*n* = 10; 8.4%; 5.8–9.5%) (Fig. 4). Frequencies of non‐mutated (expressing unique *IgG V_H_* transcripts with ≤ 3 nucleotide mutations per transcript), GC‐derived, B cells did not differ significantly between ESRD patients (*n *= 10; 6.5%; 2.0–18.8%, *P* = 0.36) and controls (*n* = 10; 4.5%; 1.4–14.0%) (Fig. 4).

## Discussion

This is the first study documenting that ESRD (also without a history of clinical auto‐immunity) is associated with elevated serum CXCL13 levels (exceeding healthy control CXCL13 levels nearly 1.5 times). This ratio was verified by comparing additional 20 ESRD patients and 20 controls (clinical and para‐clinical data not shown), showing the CXCL13 levels in ESRD to be elevated 1.6 times, *P* < 0.01. Of relevance for the CXCL13 elevations in ESRD is the fact that HD dependency is associated with a substantial increase in circulating endotoxin levels compared both to non‐dialysis‐dependent chronic kidney disease (NDD‐CKD) and to healthy controls 22. As human B cells, monocytes, macrophages, and dendritic cells respond to endotoxins with increased CXCL13 production 23, 24, 25, the elevated circulating CXCL13 levels in ESRD patients likely reflects endotoxin induced immune activation. That especially physiological changes, related to HD (i.e., increased gastrointestinal endotoxin release due to the fluid translocations occurring in the post‐dialysis period 22), may give rise to elevated CXCL‐13 levels was indicated by our observation, that CXCL13 concentrations, in 17 patients with (advanced) NDD‐CKD, did not differ significantly from those of controls but were significantly reduced compared to those of the ESRD group (data not shown). Like ESRD, NDD‐CKD is known to be associated with increased gut permeability to endotoxins 26. The lack of elevated CXCL13 levels in NDD‐CKD patients is interesting, since NDD‐CKD patients exhibit superior vaccine responses compared to HD dependent ESRD patients 27.

As a low molecular weight protein (approximately 10 kDa), CXCL‐13, like CXCL8 (IL‐8) 28, should be dialyzable. The dissociation constant K_d_, between CXCL13 and its receptor complex CXCR5/Epstein–Barr virus‐induced receptor 2 (EBI2), is 1.49 × 10^−7^ M 29. This K_d_ value is more than 20‐fold higher than circulating pre‐dialysis CXCL‐13 concentrations. Within the context of ESRD, the majority of CXCR5 receptors are therefore supposed to be free, suggesting a rapid turnover of CXCL13 in ESRD, consistent with the rather short half‐lives (minutes) of circulating cytokines in general 28. Increased endotoxin exposure has not been observed during HD therapy 22, suggesting that the HD procedure per se was not the cause for the unchanged CXCL‐13 levels (immediate) post‐dialysis. Instead, the lack of difference and the correlation between pre‐ and immediate post‐dialysis CXCL13 levels suggest, in combination with indications of rapid CXCL13 turnover and CXCL13 dialyzability, a substantial and continuous de novo CXCL‐13 production in ESRD, largely unaffected by the HD procedure itself. The elevated CXCL13 levels, in ESRD, can be viewed as an appropriate response to the immunological danger signals 30 constituted by the increased gut mediated endotoxin release, since CXCL13, through positive auto‐feedback, controls lymphoid neo‐genesis 31 and hence prepares the humoral immune system to deal with potential microbial threats. Elevated CXCL13 levels induce internalization of CXCR5 32 consistent with the tendency toward decreased CXCR5 expression in ESRD pTFH. It needs to be emphasized, that CXCL13‐induced CXCR5 internalization, within the GC, is a tightly regulated and localized process 32. Hence, dysregulated CXCL13 production, within the GC of the ESRD patient, could perturb the dynamics of GC residing CXCR5^+^ B and T lymphocytes consistent with the antibody dysregulation observed in conditions with elevated CXCL13 production as well as internalization of CXCR5 33, 34.

The moderately elevated CXCL13 levels, characterizing the ESRD group, were consistent with the general absence of patients with active autoimmunity within this group. Though upregulation of CXCL13 was implicated in the development of autoimmune insulitis in mice 35, we observed no difference in serum CXCL13 levels between ESRD patients with (primarily IDDM) and without a history of clinical autoimmunity. Opposite the pronounced CXCL13 elevations (elevated six times relative to controls) encountered in active SLE 12 and active rheumatoid arthritis 36, the long clinical history with type I diabetes, characterizing our IDDM ESRD patients (data not shown), was consistent with extinguished insulitis 37. This could explain the lack of more pronounced CXCL13 elevations in these ESRD patients.

Reduced frequencies and concentrations of CCR7^+^ CXCR5^+^ pTFH were features of ESRD. Although CXCR5^+^ CCR7^low^ PD‐1^high^ pTFH are mentioned as a subset of efficient antibody inducing blood TFH 9, previous, 8, 38, as well as a recent, reports 39 have emphasized that the phenotypic profile CCR7^+^CXCR5^+^ is sufficient to delineate true pTFH capable of helping naïve B cells. Frequencies of CD4^+^ CD45RA^−^ (memory) T cells were increased in the ESRD compared to the control group, consistent with previous reports 40 and with the pro‐inflammatory state of ESRD 41. Consequently, measured as percentage of CD4^+^ T cells, pTFH frequencies were not reduced in the ESRD group relative to controls. However, after vaccinating controls with alum‐conjugated vaccines, we observed CCR7^+^ CXCR5^+^ pTFH to be the most expanded CD4^+^ memory T cell subset (data not shown). Among the inflammatory cytokines induced by alum‐conjugated vaccines are IL‐1β and IL‐18 42. Interestingly, IL‐18 levels are also increased secondary to ESRD 43. Taking the increased IL‐18 levels and the increased infectious burden of ESRD patients into account, suggest that the reduced pTFH frequencies, among antigen experienced ESRD CD4 memory T cells, reflect compromised pTFH generation and/or survival.

ESRD pTFH were more apoptotic than control pTFH, but pTFH, in general, were less apoptotic than their CD4^+^ CXCR5^−^ memory T cell counterparts consistent with the basal inertness of pTFH 8 and consonant with an anti‐apoptotic effect mediated by CXCR5 44, 45. Hence, due to increased CXCR5 internalization, ESRD pTFH may be engendered more susceptible to the generalized lymphocyte stress encountered in ESRD 46, resulting in selective pTFH apoptosis and death.

Our protocol did not allow for vaccination. In another context, however, we observed, as previously reported 9, pTFH expansion, 1‐week post‐vaccination, in three out of four vaccine responders (not ESRD patients; data not shown). These dynamics contrasted with reduced (pre‐vaccination) pTFH frequencies and delayed (>1‐week post‐vaccination) pTFH expansion in a poor vaccine responder with a humoral immunodeficiency (data not shown). Collectively, these data support an association between pTFH and vaccination dynamics and suggest a clinical importance of diminished pTFH frequencies in ESRD. The observation that HIV patients, with a peripheral CD4^+^ CXCR5^+^ count 60 × 10^6^/L, responded significantly less to diphtheria toxoid, at week 24 47, also indicated a role for the much reduced pTFH concentrations (median: 30 × 10^6^/L) in contributing to the attenuated vaccination dynamics in ESRD 48. However, pTFH are important not only for vaccination responses but also for the dynamics of antibody recall responses in general 16. Hence, decreased pTFH levels, increased pTFH apoptosis, and potentially disturbed GC migration (due to dysregulated CXCL13 secretion and reduced CXCR5 surface expression) collectively point to slower antibody dynamics as underlying the compromised humoral immunity in ESRD.

SHM is a process which ensures additional antibody diversity and provides the basis for the subsequent selection of high‐affinity antibodies (affinity maturation). SHM correlates with the neutralizing potential of antibodies 49 as well as infectious susceptibility 50. We used primers specific for IgG in order to determine SHM in circulating isotype switched IgG^+^ memory B cells, that is, B cells which had been implicated in GC reactions. Long‐lasting high‐affinity antibody responses to protein antigens (such as hepatitis B and influenza) rely heavily upon GC reactions 51. Circulating, GC‐derived, IgG^+^ memory B cells have, also in elderly individuals, a rapid turnover. Assessed by deuterated glucose, the circulating isotype switched memory B cell subset displayed a disappearance rate (half‐life) of around 13 days 52. As our ESRD patients had minimum 6 months of HD dependence, we feel confident that the SHM data, obtained from the ESRD patients, pertained to their state of ESRD. Determination of the degree of nucleotide mutation, within the antigen‐binding domains (comprising *CDR 1* and *CDR 2*) of the *IgG V_H_* domain, demonstrated that ESRD is not accompanied by markedly reduced GC‐specific SHM (approximately 10%, compared to controls) nor by an increased frequency of non‐mutated GC‐derived B cells. Hence, our data suggest that GC‐derived antibodies/B cells, generated in the context of ESRD, have (largely and homogeneously) retained their diversity and hence the basis for the selection of high‐affinity antibodies through the process of affinity maturation. This suggests preserved neutralizing potential of the antibodies themselves, although the ability of phagocytes to opsonize may be compromised in ESRD 53. Importantly, it also provides a rationale for pursuing vaccination of ESRD patients 54 as their attenuated vaccine responses, as indicated by our data, result from altered GC dynamics and not from structural antibody deficiencies. Although SHM does not provide information on antibody dynamics and was only slightly reduced in the ESRD group, the tendency toward SHM reduction in the ESRD group was consonant with the reduced pTFH frequencies. This relates to the importance of the follicular counterparts of pTFH for the reiterative process of SHM within the GC 55. As pTFH differentiation both mirror the development of their follicular counterparts 9 and as pTFH, upon antigen reencounter, home back into the GC, in order to differentiate into their follicular cousins 9, the reduced pTFH levels were in fact consistent with a tendency toward reduced SHM in ESRD. In conclusion, ESRD is characterized by elevated circulating levels of the GC‐derived chemokine CXCL13, reduced frequencies and concentrations of pTFH, pronounced pTFH apoptosis but largely intact GC‐specific SHM. Collectively, these findings suggest that a component of the antibody deficiency, in ESRD, is primarily due to disturbed GC dynamics rather than antibody quality per se.

## Authors' Contributions

Kristian Assing designed the study and collected material. Christian Nielsen performed flow‐cytometric analyses. Marianne Jakobsen and Knud Vejen performed PCR work. Alexander Scholze provided serum samples from patients with non‐dialysis‐dependent chronic kidney disease. Mads Nybo performed biochemical analyses. Grete Soerensen organized patients on hemodialysis. Sussie Mortensen performed next‐generation sequencing. Torben Baringto: performed bioinformatic analysis of next‐generation sequencing data. Claus Bistrup designed the study. Kristian Assing wrote the paper and rest of the authors critically reviewed the manuscript.

## Conflict of Interest

The results presented in this paper have not been published previously, in whole, part or in abstract format. The authors declare no financial support or relationships that might pose a conflict of interest. Tables or figures have not been reproduced from other sources.
